# Do undergraduate psychology statistics textbooks make connections to their underlying epistemological basis?

**DOI:** 10.3389/fpsyg.2025.1641211

**Published:** 2025-09-29

**Authors:** Neal M. Kingston, Taylor D. Wilson, Rafael Quintana, Anqi Peng

**Affiliations:** ^1^Department of Educational Psychology, University of Kansas, Lawrence, KS, United States; ^2^T. Denny Sanford Harmony Institute, Arizona State University, Tempe, AZ, United States

**Keywords:** teaching statistics, undergraduate psychology statistics textbooks, epistemology, post positivism, NHST

## Abstract

Quantitative methods in psychology have been a source of controversy for decades. When misapplied or misinterpreted, they can provide a false sense of objectivity and/or lead to faulty inferences, impeding the progress of psychological research. Moreover, misunderstanding and misinterpretation of certain quantitative methods is rampant, even among trained practitioners and researchers. Epistemology is the philosophical discipline regarding how one can establish knowledge. As such, it is the foundational basis of all research methodology. This article evaluates the current state of undergraduate psychology statistics textbooks to see if they provide a proper epistemological basis necessary to support statistical reasoning. The hope is to identify opportunities to improve the methodological understanding of future generations of psychologists.

## Introduction

In the October 2014 issue of American Psychologist, Alice Eagly and Stephanie Riger reviewed ten psychology research methods in an article entitled Feminism and Psychology: Critiques of Methods and Epistemology. In their article they discussed the transition of scientific research from a positivistic to post-positivistic epistemology.

As part of their critique, Eagly and Riger gathered and analyzed 10 popular research methods texts regarding their representation of qualitative and quantitative methods. Those texts were Cozby and Bates (2011), Goodwin and Goodwin (2013), Gravetter and Forzano (2012), Leary (2012), Pelham and Blanton (2013), Salkind (2012), Shaughnessy et al. (2012), Stangor (2011), Trochim and Donnelly (2008), and White and McBurney (2013). Most of these texts acknowledged qualitative analysis, and some pointed out that subjectivity is inherent in the observational basis of quantitative methods. For example, Goodwin and Goodwin (2013, p. 10) stated:

nobody believes that scientists can separate themselves from their already existing attitudes, and to be objective does not mean to be devoid of such normal human traits. Rather, an objective observation, as the term is used in science, is simply one that can be verified by more than one observer.

Similarly, Shaughnessy et al. (2012) discussed social and cultural influences on psychological science.

Eagly and Riger concluded, “The content of the 10 methods textbooks that we examined revealed negligible attention to epistemology, and it is unlikely that many psychology researchers pay much attention to such matters, no doubt because postpositivist assumptions about science are broadly shared.” Alternatively, and perhaps more likely, the lack of attention to epistemology may be due to a lack of training of one generation of researchers and authors leading to a similar lack in subsequent generations. As Pallas (2001, p.9) noted, “…doctoral students in research universities engage with and are accountable to a relatively small number of faculty and other students.” Perhaps this limited exposure coupled with a limited understanding of epistemology leads to statistical inbreeding. To minimize this problem we argue, like others before us (Rodgers, 2010), that it would be better to explicitly address the epistemological foundations of statistical methods when introducing undergraduates (or graduates) to this subject.

As can be confirmed from the titles of the texts analyzed by Eagly and Riger, these are all research methods texts. Since the very basis of null hypothesis significance testing, which is the main focus of almost all introductory statistics texts, is intended to be post-positivistic – falsifying but never proving the null hypothesis – perhaps psychology statistics texts do a better job.

### Epistemology

Epistemology is a subdiscipline of philosophy concerned with how knowledge is established, and post positivism is the dominant epistemological approach in modern social science, including psychology^1^. Thus, by this definition, epistemological post positivism provides the foundation upon which all modern research methods must rest, including inferential statistics and the use of null hypothesis significance testing.

Auguste Comte (1798–1857), who coined the term sociology, is also credited with the creation of positivism, which he defined as the search for invariant laws of the natural and social world through observation, experimentation, and comparison. Positivism became the dominant theory of philosophy of science and epistemology through the early 20^th^ century. Logical positivism (also called neo positivism) was an extension of positivism developed by the members of the Vienna Circle^2^ between 1924 and 1936. Logical positivism focused on verifiability, positing that only statements verifiable through direct observation or logical proof were meaningful, but that the truth was out there waiting to be discovered.

Popper (1935, 1959) presented the idea that he expressed in English in his 1959 translation: “…all knowledge is provisional, conjectural, hypothetical—we can never finally prove our scientific theories, we can merely (provisionally) confirm or (conclusively) refute them…” This changed the focus of research from verifiability (under positivism and logical positivism) to falsifiability and is the basis of post-positivism, which became more and more prominent as the epistemological basis for empirical research since that time. The key elements underlying post-positivistic epistemology are as follows.

1. Critical realism. Reality exists independent of our perceptions, but our understanding of reality is partial and subject to revision (Fox, 2008).
2. Falsifiability (as described above).
3. Subjectivity of observation. Observations are influenced by social, cultural, and historical contexts, including the theoretical frameworks and assumptions of the observer (Mat Roni et al., 2020, p.8; Maksimović and Evtimov, 2023).
4. Probabilistic and tentative knowledge. Knowledge claims exist within certain boundaries with a certain probability (Reed, 2023). Progress in a line of research can lead to boundaries being reduced or the probability of truth lying within boundaries can be increased.

Passmore (1967, p. 56) declared “Logical positivism is dead, or as dead as a philosophical movement ever becomes.” Popper (1974, p87) declared that logical positivism was dead, and he took credit for its death. While logical positivism was dead to most philosophers, it held on as the epistemological basis of social science research methods a bit longer. Some of the erosion of the former hold possessed by logical positivism occurred hand in glove with the expansion of qualitative analysis methods. That is, the influence of post positivism in philosophy of science supported a resurgence and expansion of qualitative methodology that embraced the subjectivity and uncertainty central to post positivism. This brought greater attention to said subjectivity and uncertainty to quantitative social science researchers who often focused on the illusion of absolute objectivity in applying their methods. Quantitative economist Donald McCloskey (1989) described this phenomenon in his article, “Why I am no longer a positivist.” Now, as the death of logical positivism spread from philosophy to social science methodology, post positivism has been recognized by most experts as the epistemological basis for all social science research.

### Relationship between post-positivism and null hypothesis significance testing

Beginning even before Popper, the field of statistics started evolving in ways that had much in common with post-positivism. Starting with several papers in the early 1920s (Fisher, 1921, 1922a, 1922b, 1922c, 1924) and culminating with his text, *Statistical Methods for Research Workers* Fisher (1925), laid out the foundations of significance testing and “…almost single-handedly created the foundations for modern statistical science…” (Hald, 1998, p. 738). Soon after, Neyman and Pearson (1928, 1933a, 1933b) developed their framework for hypothesis testing. While there were similarities between the two approaches, there were also significant theoretical and practical differences. For example, Fisher’s significance testing was set up to provide one source of evidence that should be combined with other evidence. Although Fisher originally recommended dichotomous inferences based on a comparison with a critical value (such as 0.05), he eventually recommended presenting exact significance values to serve that evidentiary purpose. Neyman-Pearson, on the other hand, recommended clear *a priori* decision rules for α and β (Type I and Type II error rates). Fisher’s approach focused on making inferences based on falsifying the null hypothesis with no reference to alternative hypotheses. Neyman and Pearson required an alternative hypothesis and a consideration of Type II error, the probability that the null hypothesis is not rejected when a specific alternative hypothesis is true (Olsson and Galesic, 2011). Eventually both approaches were combined into what is the current form of null hypothesis significance testing (NHST; Schneider, 2015) and this topic is a central focus of most introductory psychology statistics texts.

In line with post-positivism, NHST is based on falsifiability – one can reject the null hypothesis, but you never accept it or any particular alternative hypothesis. In addition, most introductory psychology statistics present confidence intervals, which lead to the same inferences one might make using NHST, but present additional information, emphasizing a range of plausible values that cannot be falsified based on the current data. Importantly, this latter consideration is consistent with a post-positivistic emphasis on knowledge being tentative and probabilistic.

While there is an extensive literature criticizing null hypothesis significance testing, we will not rehash that here as it is outside the intent of this article, which is whether introductory statistics texts provide an epistemological foundation. Similarly, we will not address Bayesian approaches as they are rarely mentioned in introductory texts, though perhaps they should be.

### Common misunderstandings of inferential statistics

Inferential statistics is a cornerstone of scientific inquiry, enabling researchers to draw conclusions about populations based on sample data. Despite its importance, inferential statistics is frequently misunderstood by students, researchers, and even seasoned professionals. These misunderstandings can lead to flawed interpretations, misguided decisions, and compromised scientific integrity.

Nuijten et al. (2016) found that roughly half of all published empirical psychology articles using NHST contained at least one inconsistent *p*-value. Moreover, around one in eight articles contained a gross inconsistency that may have affected the conclusion of the study. For example, where the reported *p*-value was significant and the computed *p*-value was not, or vice versa. Hoekstra et al. (2014) showed that in more than half of a sample of published articles, a nonsignificant outcome was erroneously interpreted as proof for the absence of an effect. In about 20% of the articles, a significant finding was considered absolute proof of the existence of an effect. Based on 281 articles, Bakker and Wicherts (2011) found that around 18% of statistical results in the psychological literature were incorrectly reported.

Empirical studies have systematically documented misconceptions in the process of statistical inference, offering insights into their origins and suggesting pathways for improved statistical education. Several common areas of misconception follow.

#### Misinterpretation of *p*-values

One of the most pervasive misunderstandings of inferential statistics involves the interpretation of *p*-values. Many researchers erroneously believe that a *p*-value indicates the probability that the null hypothesis is true or that it reflects the likelihood that the observed results occurred by chance. In reality, the *p*-value represents the probability of obtaining results at least as extreme as those observed, assuming the null hypothesis is true.

An empirical study by Lyu et al. (2018) surveyed 362 psychology researchers and students, revealing that 99% misinterpreted at least one statement about *p*-values. These misinterpretations included believing that a *p*-value of 0.01 means there is a 1% chance the null hypothesis is true, or that the result is practically significant. Such errors contribute to practices like “p-hacking” and the overemphasis on statistical significance at the expense of practical relevance.

#### Misunderstanding confidence intervals

Confidence intervals (CIs) are another area rife with confusion. Many people performing statistical analyses interpret a 95% CI as meaning there is a 95% probability that the true parameter lies within the interval, which is incorrect. The correct interpretation is that if the same study were repeated many times, 95% of the calculated intervals would contain the true parameter^3^. In the aforementioned study by Lyu et al., 93% of participants misinterpreted at least one statement about confidence intervals. In a similar study of 120 researchers and 442 undergraduate students (Hoekstra et al., 2014), on average both groups endorsed more than half of a list of six incorrect statements about CIs. These misconceptions can lead to overconfidence in results and miscommunication of uncertainty in scientific findings.

#### Confusion between sample and population

Another foundational misunderstanding involves the relationship between samples and populations. Students often view samples as miniature replicas of populations rather than probabilistic representations. This misconception undermines the logic of statistical inference, which relies on sampling distributions and variability across samples. Kula and Koçer (2020) argue that this confusion stems from the way inferential statistics is taught. They distinguish between two logics: the logic of construction, which starts from the population and builds the inference framework, and the logic of application, which begins with the sample and applies statistical procedures. Most teaching emphasizes the latter, skipping over the conceptual foundations that help students understand why inference works.

#### Misunderstanding sampling distributions

Sampling distributions are central to inferential statistics, yet they are poorly understood. Students often confuse a single sample with the distribution of all possible samples, or conflate the Law of Large Numbers with the Central Limit Theorem. These misunderstandings hinder their ability to grasp concepts like standard error and the rationale behind hypothesis testing. Empirical studies cited by Kula and Koçer (2020) show that even high-achieving students struggle with these ideas. For example, many believe that a sample is a quasi-proportional small-scale version of the population, which interferes with their understanding of sampling variability.

#### Overreliance on null hypothesis significance testing (NHST)

NHST is often taught in a mechanical, almost ritualistic manner, where students mislearn to equate statistical significance with truth (Gliner et al., 2002; Sedgwick, 2023). Subsequently, researchers often neglect effect sizes, confidence intervals, and the broader context of findings. Lyu et al. demonstrated that the format in which results are presented (NHST vs. CI) significantly affects interpretation. Participants were more likely to perceive results as consistent and meaningful when presented with CIs rather than *p*-values. This does not mean that even when taught these neglected topics are taught well and diminish misinterpretations, but it does open the possibility that alternative approaches may foster better understanding and more nuanced interpretations.

One next step would be to gather evidence as to whether current textbooks provide an epistemological foundation for statistics. That is the intent of this article. If they do not, a subsequent step would be empirical research to determine if such a foundation reduces misunderstandings and improves the quality of psychology research as we suspect it might.

## Methods

The five undergraduate psychology statistics textbooks with the largest sales volumes per Amazon as of December 2020 were identified. To broaden the sampling frame, five Carnegie classified Research 1 (Kosar and Scott, 2018) universities (three public, two private) with large undergraduate psychology enrollments were selected to ensure geographical (one west coast, one southwest, one midwest, and two east coast) diversity and the textbook each used for their introductory psychology statistics course was also identified. Two of those schools, Arizona State University and University of Virginia used the same text, so our analysis was based on nine textbooks. Table 1 presents titles, citations, and the name of that institution, for each of the textbooks.

**Table 1:** Nine textbooks examined for explicit references to post-positivistic epistemology.

Title	Citation
Essentials of Statistics for the Behavior Sciences (10th ed.)	Gravetter et al. (2020)
Statistics for the Behavioral Sciences (10th ed.)	Gravetter and Wallnau (2016)
Basic Statistics for the Behavioral Sciences (7th ed.)	Heiman (2013)
Statistics for the Behavioral Sciences (3rd ed.)	Privitera (2017)
Fundamental Statistics for the Behavioral Sciences (9th ed.)	Howell (2016)
Essentials of Statistics for the Behavioral Sciences (5th ed.)^1^	Nolan and Heinzen (2020)
Statistics for Psychology (6th ed.)^2^	Aron et al. (2012)
Introduction to the New Statistics: Estimation, Open Science, and Beyond^3^	Cumming and Calin-Jageman (2016)
Statistics for the Behavioral Sciences (5th ed.)^4^	Nolan and Heinzen (2020)

^1^ University of Kansas. ^2^ Arizona State University, University of Virginia. ^3^ University of Southern California. ^4^ Columbia University.

Publishers of each textbook were contacted, and we requested an electronic version of each textbook. In advance of our analysis, we chose 41 search terms that we believed would allow us to find sections of the texts that directly or indirectly addressed any of the following *a priori* identified themes that might indicate an explicit or implicit connection between statistics and epistemology: (1) research and statistical analysis have an epistemological basis, (2) research is based on falsifiability, (3) research findings may not be universal, and (4) research results contain uncertainty, and (5) all research has subjective aspects (both in terms of participants and the research team). The search terms were selected by the authors to represent different possible ways to find these sections of texts and included words related to subjectivity and culture (e.g., constructivist, country, culture, occidental), falsification (e.g., falsification, hypothesis test, null hypothesis), truth (e.g., argument, fact, generalization, impossible, infallible, universal), and sampling (e.g., population, subgroup). The complete list of search terms is presented in Table 2.

**Table 2:** Search terms.

argument	history	neutral	population
behavior	human nature	never	prove
behaviorism	humans	norms	realism
constructivist	hypothesis test	null hypothesis	subgroup
country	hypothetico-deductive	objectivity	truth
culture	impossible	observation	universal
empiricism	infallible	occidental	value
fact	invariance	opinion	value-free
falsification	law	parameter	value-laden
generalization	nature	pluralism	world

Each term was used to search through the text and when found, the text was read for about one page before and after the instance that was found. Any text that was connected to epistemology was noted. To be clear, the purpose of the analysis was not to find these specific words, but instead to use those words to find sections of the text that might be related to epistemology.

## Results

Searching through the nine textbooks using the keywords led to variation around the aforementioned five themes.

### Theme 1: epistemological basis

None of the texts mentioned the terms positivism, logical positivism, or post-positivism. We could find no explicit mention of any connection between statistics and epistemology.

### Theme 2: falsifiability

Five of the nine textbooks (e.g., Cumming and Calin-Jageman, 2016; Gravetter and Wallnau, 2016; Heiman, 2013; Howell, 2016; Privitera, 2017) explicitly pointed out that researchers can ***never*** prove whether the null hypothesis is true. For example, in the textbook of *Basic Statistics for the Behavioral Sciences (7^th^ edition)*, Heiman (2013) wrote that “…we can never prove whether the null hypothesis is true” (p. 216). Gravetter and Wallnau (2016) wrote in the textbook of *Statistics for the Behavioral Sciences (10^th^ edition)* that “It is impossible to prove that H_0_ is correct…” (p. 262). Similarly, in the textbook *Statistics for the Behavioral Science*, Privitera (2017) also mentioned that it is not possible to prove the null hypothesis. As the null hypothesis is impossible to prove, researchers need to be careful when they interpret the results. Two other textbooks (Aron et al., 2012; Nolan and Heinzen, 2020) indicate that when researchers do not reject the null hypothesis, they cannot say the null hypothesis is accepted or proved. In other words, it is inappropriate to conclude that there is no difference or treatment effect because failing to reject H_0_ means there is not enough evidence to prove there is a difference or an effect. Nolan and Heinzen (2020) wrote that “There might be a real mean difference that is not extreme enough to be picked up by the hypothesis test. We just cannot know.” (p. 206). Likewise, Heiman (2013) mentioned “…we have not proven that H_0_ is true, so we have not proven that our independent variable does not work. We have simply failed to find convincing evidence that it does work.” (p.222). On the other hand, when researchers decide to reject the null hypothesis, they could report the results are statistically significant or support the research hypothesis instead of accepting the alternative hypothesis (H_1_). For example, in the textbook of *Essentials of Statistics for the Behavioral Sciences (10^th^ edition)*, Gravetter et al. (2020) wrote that “We do not state there is an effect. Instead, we state there is evidence for an effect. The distinction is subtle but important. By rejecting the null hypothesis, we are not proving the existence of a treatment effect (that is, we are not proving the alternative hypothesis to be true).” (p. 253).

### Theme 3: uncertainty

The majority of the textbooks mentioned that in quantitative research, there is always uncertainty. In other words, statistics are observations that contain sampling error, which indicates that they are not perfect and infallible. For example, in the preface of the textbook *Statistics for Psychology (6^th^ edition)*, Aron et al. (2012) mentioned that “…statistics are not “given” by nature, not infallible, not perfect descriptions of the events they try to describe….” In the same textbook, on page 89, the authors mentioned “…scientific research of any kind can only make that truth or effectiveness seem more or less likely; it cannot give us the luxury of knowing for certain.” Similarly, in the textbook of *Statistics for the Behavioral Sciences (5^th^ edition)*, Nolan and Heinzen (2020) showed that “…researchers never know whether they are correct or incorrect” (p.135) and “One problem with this analytical approach is that we do not have direct access to the truth about what we are studying. Instead, we make inferences based on the data we collected. Our decision could be right or wrong. But, a researcher’s goal is to be correct as often as possible.” (p.235). Thus, when researchers test hypotheses, they can only approach the truth but can never fully achieve the truth. Other sources of uncertainty were mentioned less frequently, if at all (for example, see the discussion of theme 5).

### Theme 4: non-universality

Multiple textbooks pointed out that statistical results might not be universal. In other words, research findings based on a single study cannot be generalized to a broader population than the one from which the original subjects were drawn. They provided several reasons. For example, Heiman (2013) and Gravetter et al. (2020) mentioned that researchers cannot be certain that there is a difference or treatment effect in the population which is too large to be measured. “…we need inferential statistics because there is no guarantee that the sample accurately reflects the population. In other words, we are never certain that a sample is representative.” (Heiman, 2013; p. 195). Similarly, when it comes to the null hypothesis, “There are many ways in which a real mean difference in the population might not be picked up by a sample.” (Nolan and Heinzen, 2020; p. 132). Thus, if researchers collect data with a large group of representative participants, they will increase their confidence to make an accurate observation and approach the truth.

### Theme 5: subjectivity and the role of human experience

There is only one textbook that mentioned the important role of human experience in the research study “…quantitative researchers jump to conclusions about the phenomenon without first exploring the human experience of it through free-response interviews or observation.” (Aron et al., 2012; p. 53).

In summary:

- None of the texts mentioned positivism, logical positivism, or post-positivism or made any explicit connection to epistemology.
- Five of nine textbooks explicitly and strongly stated that researchers can never prove the null hypothesis is true, and two others stated so somewhat less emphatically;
- Most, but not all, of the texts discussed the idea that statistics can never prove anything with certainty;
- Five of nine textbooks explicitly stated that a sample might not be representative of the population of interest;
- Only one textbook explicitly stated the importance of the human context surrounding the research study.

## Discussion

In the literature review, we demonstrated that both students and professionals often do not understand inferential statistics. In our analysis of nine introductory statistics texts we showed that like the research methods textbooks analyzed by Eagly and Riger (2014), introductory psychology statistics textbooks pay almost no explicit attention to epistemology and little implicit attention. Perhaps these two findings are connected. It seems self-evident that if students understood the falsifiability paradigm that they would be less likely to believe the null hypothesis (or any other hypothesis) was verified when an analysis did not reject the null hypothesis.

On the other hand, there is a small body of literature that might support the status quo. Corrado Matta (2022) pointed out that, “Introducing philosophical paradigms without clear pedagogical framing risks overwhelming students and obscuring methodological understanding.” Perhaps the necessary scaffolding would leave insufficient room for other objectives or would produce cognitive overload that interferes with learning. Alternatively, perhaps undergraduate psychology students, many of whom have little interest in the quantitative aspects of the field, might be further disengaged by the addition of a layer of philosophy.

Given the rampant misconceptions regarding statistical inference, we are not convinced by these counter arguments, but neither can we assume that addressing epistemology will reduce these misunderstandings. We recommend that the impact of the teaching of the epistemological bases of inferential statistics be evaluated to see if this improves student learning.

### Caveats

This study was based on only nine texts, all of which are written in American English. Results might not be the same for texts written in other languages, in other countries, or aimed at other disciplines. Texts were published between 2012 and 2020 and there appears to be no pattern based on year of publication.

Following, are some recommendations for textbook authors (or an instructor stuck with a text that does not make these points!):

- Every textbook on statistics (and research methods, for that matter) should discuss its epistemological basis in the introductory chapter and reinforce those concepts throughout.
- Discuss issues of subjectivity (both of the participants being studied and the researchers) and context in the framing of research questions, collecting data, and data analysis.
- Dedicate sections to explaining and correcting pervasive misunderstandings –such as misinterpretations of *p*-values and confidence intervals.
- Focus on confidence intervals instead of, or at least in addition to, null hypothesis significance testing, since the very nature of the confidence interval reinforces that there is not a single true value that we have determined.

With these suggestions we hope to bolster the methodological understanding of future researchers.

## Data Availability

The datasets presented in this article are not readily available because they are copyrighted by their original publishers. Further inquiries can be directed to the corresponding authors.

## Footnotes

^1^We note that there are many modern epistemological approaches besides post-positivism. Some of these overlap with post-positivism while maintaining some distinct differences. Because of the relationship between post-positivism’s focus on falsifiability and approaching but never achieving absolute truth, and the relationship this idea has to inductive statistics, we have chosen to focus solely on post positivism in this article.

^2^The Vienna Circle was a group of renowned philosophers, scientists, and mathematicians who periodically met in Vienna to form a sounder basis for scientific methods. The group was chaired by philosopher/physicist Moritz Schlick and had more than 30 members across its active years.

^3^An additional interpretive nuance distinguishes between probabilities assigned to confidence intervals before and after the data from an experiment are observed. The interested reader is referred to Mayo (1981).
